# Oviposition site attraction of *Aedes albopictus* to sites with conspecific and heterospecific larvae during an ongoing invasion in Medellín, Colombia

**DOI:** 10.1186/s13071-019-3710-6

**Published:** 2019-09-18

**Authors:** Talya Shragai, Laura Harrington, Catalina Alfonso-Parra, Frank Avila

**Affiliations:** 1 Department of Entomology, Cornell University, Ithaca, NY USA; 2 Instituto Colombiano de Medicina Tropical, Universidad CES, Sabaneta, 055450 Antioquia Colombia; 3 Max Planck Tandem Group in Mosquito Reproductive Biology, Universidad de Antioquia, Medellín, 050010 Antioquia Colombia

**Keywords:** *Aedes aegypti*, *Aedes albopictus*, Interspecific competition, Oviposition choice, Mark-release-Recapture, Semi-field cage

## Abstract

**Background:**

*Aedes aegypti* and *Aedes albopictus* are two globally invasive vectors with similar ecological niches. Encounters between them can result in either competitive exclusion or stable co-existence, but it is unclear what drives these variable outcomes. Larval competition in favor of *Ae. albopictus* is a main hypothesis for the competitive exclusion of *Ae. aegypti* observed in some regions. However, the role of oviposition preference in determining the degree of competitive larval interactions in the field is not well understood. In this study, we used a combination of mark-release-recapture methods with ovitraps in the open-field and a semi-field cage to test whether gravid *Ae. albopictus* seek oviposition sites in response to the presence, species, and density of either conspecific or heterospecific *Ae. aegypti* larvae in the aquatic habitat. We conducted our study in Medellín, Colombia, where *Ae. aegypti* is a long-term resident and *Ae. albopictus* is a recent invader.

**Results:**

In the open-field and semi-field cage experiments, gravid *Ae. albopictus* showed strong preference for ovitraps with larvae over those without. They consistently preferred ovitraps with higher density of conspecific (*Ae. albopictus*) larvae and low density of heterospecific (*Ae. aegypti*) larvae over traps with no larvae or high density of heterospecific (*Ae. aegypti*) larvae. In the semi-field cage experiment, traps with low density of *Ae. albopictus* were not preferred more or less than any other trap, but in the open-field experiment they were preferred over traps without larvae.

**Conclusions:**

We demonstrate, through open-field and semi-field cage experiments, that *Ae. albopictus* are more attracted to oviposition sites with larvae and that the combination of species and density of larvae influence attraction. This demonstrated preference could increase interspecific larval competition as *Ae. albopictus* actively seek containers with conspecific and heterospecific larvae. Any resulting competition with *Ae. aegypti* may favor one species over the other and alter the distribution or abundance of both. Because these species vary in vectorial capacity and insecticide resistance, effects of interspecific competition could ultimately impact arbovirus transmission rates and the success of vector control efforts
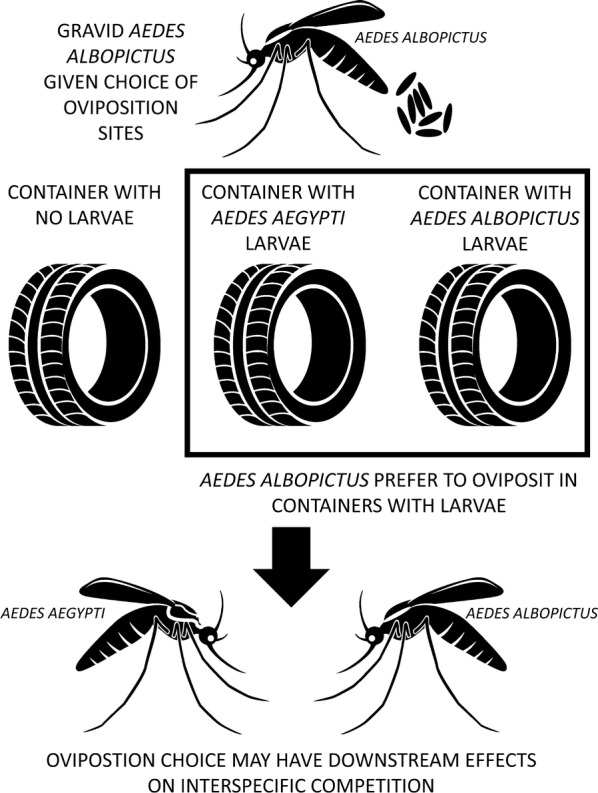
.

## Background

*Aedes aegypti* and *Aedes albopictus* mosquitoes are two highly successful invasive species that transmit the most important arboviruses impacting human health, including dengue, yellow fever, Zika and chikungunya. *Aedes aegypti* has spread throughout the global tropics, while *Ae. albopictus* has colonized every continent except Antarctica [1]. *Aedes albopictus* is currently invading Medellín, Colombia, where *Ae. aegypti* is well established, and both species have recently begun to coexist in some urban regions of Medellín [2]. Both *Ae. albopictus* and *Ae. aegypti* deposit their eggs above the water line in natural and artificial containers, thrive in urban/peri-urban environments, and are readily anthropophagic [3, 4]. Their overlapping ecological niches result in high encounter rates; interestingly, the results of these interactions vary. In some cases, species overlap results in competitive replacement of *Ae. aegypti* by *Ae. albopictus* [5, 6], while the reverse can also occur [7], and at times these two species stabilize and coexist [8].

The long-term outcome of co-occurrence on the distribution and abundance of each species has significant public health impacts. *Aedes aegypti* and *Ae. albopictus* differ in their feeding behavior [9] and their ability to transmit pathogens and parasites [10], meaning a shift in species abundance could alter the spread or intensity of arboviral disease. *Aedes aegypti* and *Ae. albopictus* also vary in insecticide resistance status [11], so efficient control strategies rely on understanding species distribution. Increased clarification of the ecological drivers behind competitive dynamics will enable more accurate predictions for the spread of these vectors, which will in turn allow for better-informed disease mitigation.

There are many hypotheses for why coexistence *versus* competitive exclusion may occur [12]. Larval competition is the most cited and commonly tested theory, and almost all studies agree that, when forced to share the same aquatic habitat, larval *Ae. albopictus* outcompete *Ae. aegypti* [5, 13]. However, because interspecific encounters do not always result in competitive exclusion of *Ae. aegypti* [7, 8], other determinants must be at play.

One understudied factor in larval competition is the role of mosquito oviposition behavior. In order for larval competition to favor *Ae. albopictus* over *Ae. aegypti*, the two species must first deposit their eggs (oviposit) in the same containers. Mosquitoes do not choose oviposition sites at random; rather, they respond to multimodal cues to select preferred habitat [14–17]. If *Ae. albopictus* actively seek or avoid oviposition sites based on the presence or absence of *Ae. aegypti* or *Ae. albopictus* larvae, this would increase or decrease the degree of competition. However, experiments to date have mostly been laboratory-based [18, 19] or observational [7, 20]. There is one published open-field-based study on how oviposition site preference can affect interspecific larval competition. This study collected naturally occurring eggs in containers with water used to previously rear varying levels of pre-existing conspecific and heterospecific larval density [21], and found evidence that these factors can drive *Ae. albopictus* oviposition site usage. However, the direction of this preference was unclear.

We conducted an experiment to test the hypothesis that *Ae. albopictus* oviposition site attraction in Medellín Medellín, Colombia, is driven by the presence, density, and species of either conspecific or heterospecific *Ae. aegypti* larvae in the aquatic habitat. While laboratory experiments control for environmental variability, they cannot always be extrapolated to the field, and while observational studies and natural collections report on true conditions, their design makes it difficult to pinpoint the ultimate cause of observed patterns. A combination of semi-field cage trials conducted under natural environmental conditions and open-field experiments with laboratory-reared cohorts of ovipositing females can maximize experimental control without losing real-world applicability. To this end, we first tested this hypothesis in the open-field using mark-release-recapture, and then replicated the experiment under semi-field cage conditions.

## Methods

### General methods

#### Study location

**Fig. 1:**
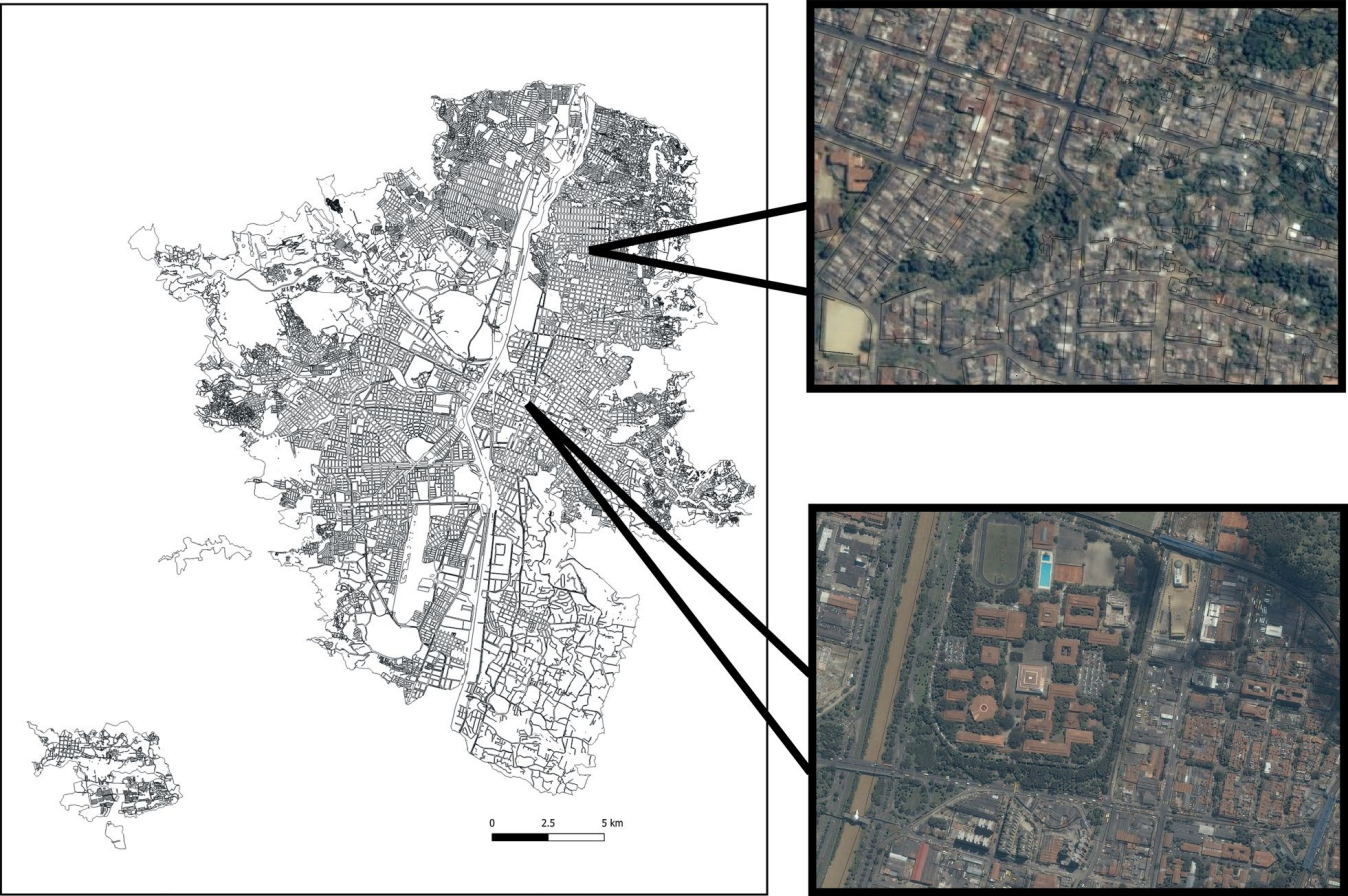
Map of Medellín with close up satellite imagery showing the location of the field site (top) and semi-field cage site (bottom)

We conducted the semi-field cage experiment on the Universidad de Antioquia campus, 6°16ʹ4.97″, − 75°34ʹ7.91″ and the open-field experiment in Barrio Santa Cruz, Medellín, Antioquia, Colombia, 6°17ʹ44.42″, − 75°33ʹ11.76″ (Fig. 1). *Aedes aegypti* has been established in both neighborhoods over a long period of time while *Ae. albopictus* was first detected through systematic city-wide ovitrap surveillance in Barrio Santa Cruz and in Barrio Universidad de Antioquia in 2011 (Secretaria de Salud Medellín, unpublished data). During the month-long semi-field cage study and the three-month open-field study, the average Medellín temperature was 21.6 °C and the average rainfall was 119 mm. Santa Cruz is a dense urban residential community in the steep hills in the northeast of Medellín, with a population of 430 inhabitants per hectare. The government of Medellín categorizes 96.6% of the Santa Cruz population as socioeconomic status 2 and 3.4% as status 1 based on a ranking system of 1 to 6, with 1 being the lowest [22]. Most homes in Santa Cruz are open to the outdoors, without screens.

#### Container survey and selection of release houses

For this experiment to reflect accurate field conditions and to select release houses that were already positive for both species, we first surveyed naturally occurring larvae in Santa Cruz and determined the locations of oviposition sites. Although both can thrive indoors and outdoors, *Ae. aegypti* preferentially rest, feed, mate, and oviposit inside [23], while *Ae. albopictus* tend to be exophagic and exophilic [3]. Different cities have very different degrees of access to indoor/outdoor habitats and availability of indoor/outdoor oviposition sites, and this largely determines if larval habitat is located inside or outside the house, as in Moore et al. [24]. After obtaining permission from homeowners through door-to-door interviews, trained field personnel surveyed the indoor and outdoor areas of each house. Teams identified all water-holding containers suitable for larval development (defined by ability to hold at least 2 ml of water and residents reported that they did not regularly clean and change the water) and recorded container location (indoor or outdoor) and presence or absence of mosquito larvae/pupae. Larval/pupal collection and species identification was as described in Shragai et al. [25].

We conducted the experimental release studies at three houses within Santa Cruz that were selected based on the following criteria: (i) full consent of the homeowner(s) was given; (ii) each contained outdoor space on the property; and (iii) each was found to have natural populations of both *Ae. aegypti* and *Ae. albopictus*, detected both as larvae and adults. Before each release, each home and all adjacent homes were thoroughly cleared of any potential oviposition sites.

#### Mosquitoes and oviposition traps preparation

*Aedes albopictus* used in the study were of low generation (F6–F8) adults from a colony established with field-collected larvae in Medellín, Antioquia, Colombia. *Aedes aegypti* used in the ovitraps were of similar low generation (F6–F8) *Wolbachia-*negative larvae from a colony established from Acacías, Meta, Colombia. These were known to be *Wolbachia-*negative because no *Wolbachia* releases have been conducted in Meta, Colombia. We did not release *Ae. aegypti* in this study.

All mosquitoes were hatched and reared as described previously (Alfonso-Parra et al. [26]. Released females were offered a blood meal from author Talya Shragai (TS) on days 5 and 6 post-eclosion, and all visibly engorged females were transferred to a separate cage provided with water, 10% sucrose *ad libitum*, and a daily human blood meal for 5 days until fully gravid.

One day post-hatch and four days pre-release, first-instar larvae were placed in the experimental ovitraps. The ovitraps were filled with 3.7 l of tap water at least 24 h prior to the addition of larvae to allow chlorine to dissipate. Experimental ovitraps contained two ground Hikari Gold Cichlid food pellets (mean weight of 3.56 g/pellet) (Hikari, Himeju, Japan) and one of two levels of larval density low density (20 larvae, density of 1 larva per 185 ml) or high density (100 larvae, density of 1 larva per 37 ml) of *Ae. aegypti* or *Ae. albopictus.* This nutrition level was chosen to be in surplus for both density treatments in order to equalize larval development rates and isolate preference based on signals received from healthy, non-stressed larvae.

#### Trap design and experimental conditions

**Fig. 2:**
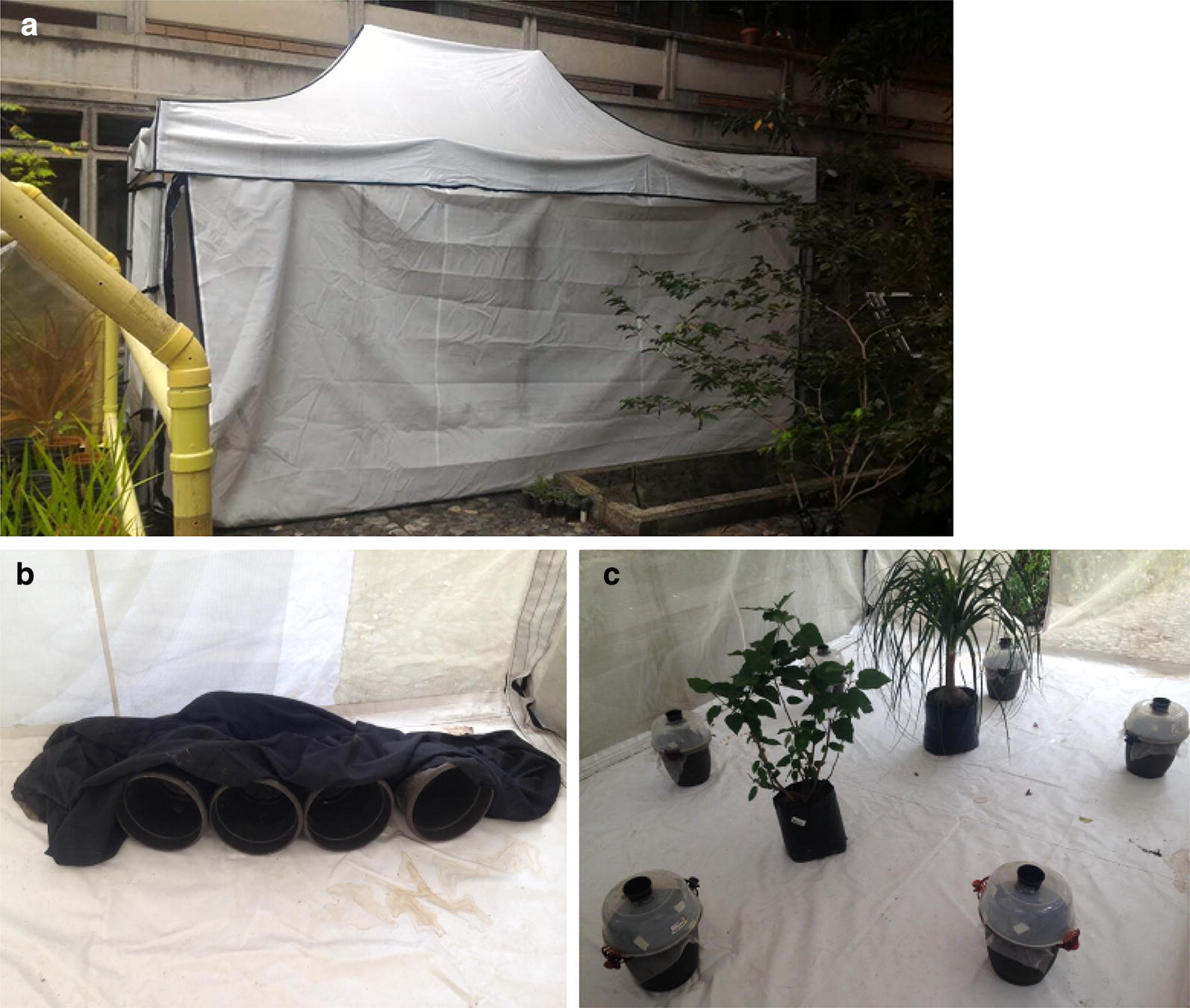
Set-up of the semi-field cage. **a** Field cage exterior. **b** Field cage interior, showing close-up view of buckets with cloth. **c** Field cage interior, showing placement of ovitraps and resting sites: buckets with cloth and plants

Gravid females were recaptured using modified sticky ovitraps (Fig. 2c). Ovitraps were constructed from 11.4 l black plastic buckets. The buckets were covered with mesh to ensure that no mosquitoes could escape if they eclosed in the trap and to prevent any mosquitoes from successfully laying eggs in the traps and potentially affecting semiochemicals in the oviposition water. A 1.9 l clear plastic food storage container with a 7.6 cm hole cut into the center bottom was fitted upside down on top of the bucket, and the sides of the plastic container were lined with clear plastic sheeting coated in Tangle Trap (Scotts Miracle-Gro, Marysville, OH). A 0.4 l black plastic cup with the bottom removed was fitted into the hole in the clear plastic container. Each experimental block included four ovitraps representing each larval density-species combination and two control ovitraps that contained water and fish food but no larvae.

### Open-field experiment

To test if *Ae. albopictus* attraction to oviposition sites is driven by the presence, density, and species of either conspecific or heterospecific *Ae. aegypti* larvae in an open field environment, we conducted a series of mark-release-recaptures using sticky ovitraps seeded with varying densities and species of larvae.

#### Mark-release-recapture

We marked mosquitoes on the day of release with DayGlo (DayGlo Color, Cleveland, OH, USA) fluorescent dust following the methods of Edman et al. [27], Harrington et al. [28], and others (Fig. 2). This method does not affect the fitness of the mosquitoes; most mosquitoes are marked for life (Harrington, unpublished data). We used a different color dust for each release site to quickly differentiate location of origin.

We conducted eight rounds of releases between 12 June and 8 August 2018. Mosquitoes were transferred to 473 ml plastic cups for transportation to the release sites. Gravid females were released in the late afternoon (15:00–17:00 h), adjacent to ovitraps. Between 25 and 45 females were released in each of three houses in each round as described above.

Gravid females were recaptured using the sticky ovitraps described above, using the same experimental species and density conditions. Based on the results from the container survey, ovitraps were placed in the backyard of each release house in the location best representing natural *Ae. albopictus* larval habitat in urban residential Medellín. Ovitraps were checked every 24 h for four days starting 24 h after release. All captured mosquitoes were examined in the field to record species, sex, and presence/absence of fluorescent dust, and mosquitoes were then removed and discarded.

### Semi-field cage experiment

To test if *Ae. albopictus* attraction to oviposition sites is driven by the presence, density, and species of either conspecific or heterospecific *Ae. aegypti* larvae under more highly controlled conditions, we replicated our open-field experiment in a semi-field cage.

#### Semi-field cage

The semi-field cage consisted of a 3.0 × 4.5 × 1.9 m (w × l × h) tent cage (Fig. 2a) made of white PVC coated polyester mesh, reinforced at angles and seams with white canvas tarp and supported by an aluminum frame. The cage was accessed using a front zipper door. The floor of the cage was made of white plastic for easy visualization of mosquitoes. The cage was placed under full tree cover to protect it from direct sunlight. The following items were placed inside the cage to provide refugia for mosquitoes (Fig. 2b, c): two potted plants (*Beaucarnea* sp., and *Hibiscus* sp.), four dry plastic buckets resting sideways, and a black cloth. Plants were watered as needed and leaves were misted daily to provide drinking water for released mosquitoes.

Ovitraps were placed in a circle around the center of the semi-field cage. The order of ovitraps was kept the same throughout the experiment, but the position was rotated one place clockwise between each replicate to identify any positional bias.

#### Release-recapture

We conducted four different releases between 19 January and 19 February 2019. Females were released in the afternoon (13:00–16:00 h), in the center of the semi-field cage. Between 22 and 62 females were released in each replicate. Mosquitoes that did not voluntarily fly from their container when the lid was opened and the side tapped, were transported back to the laboratory and were not counted in the total number released. A point temperature reading both inside and outside the semi-field cage was taken when the mosquitoes were released and on each recapture day to ensure that temperatures inside the cage accurately reflected environmental conditions.

Ovitraps were checked every 24 h for two days starting 24 h after release. All captured mosquitoes were removed and discarded. Any remaining uncaptured mosquitoes were killed with an electric racket at the end of each replicate (Black Flag, Madison, WI, USA).

### Data analysis

The open-field data and semi-field cage data were analyzed individually using separate models. For both, we used a generalized linear mixed model using Poisson distributions to analyze the effects of ovitrap treatment on *Ae. albopictus* recapture. Date of release and ovitrap ID were included as random factors and for the open-field experiment, house of release was included as a blocking factor. The number of *Ae. albopictus* recaptured over the experiment was used as the dependent variable, and four forms of fixed effects structures were used: larvae/no larvae, species, density, and each of the five species-density combinations. For the response variable, the number recaptured for all days in each trap of each release replicate was totaled. Further pairwise analysis was conducted by calculating the estimated marginal means and performing all pairwise comparisons. All analyses were conducted in R (R Core Team, Version 3.5.2), using the *lme4* and *emmeans* packages [29–31].

Although we collected wild, non-marked *Ae. albopictus* in the open-field study, we did not include an analysis of these mosquitoes because the sample size (total *n* = 35) was too low to detect statistical differences.

## Results

### Open-field experiment

#### Container survey

Only 9.68% of the houses surveyed had at least one positive container (Table 1). Overall, 83.17% of 305 containers surveyed were negative and 16.83% were positive. Of the positive containers, 72.55% were positive for *Ae. aegypti* alone, 19.61% were positive for *Ae. aegypti* and *Ae. albopictus*, and 7.84% were positive for *Ae. albopictus* alone. Almost all containers identified in the survey, including those both positive and negative for larvae, were located indoors.

**Table 1:** Container survey results. Containers and houses negative and positive for *Ae. aegypti* and *Ae. albopictus*. Results are divided into containers and houses with containers located indoors and outdoors

	Containers surveyed	Houses surveyed
Indoors		
Positive, *Ae. aegypti*	8	2
Positive, *Ae. albopictus*	0	0
Positive, *Ae. aegypti* and *Ae*. *albopictus*	0	0
Negative	19	370
Total	27	372
Outdoors		
Positive, *Ae. aegypti*	29	28
Positive, *Ae. albopictus*	4	2
Positive, *Ae. aegypti* and *Ae*. *albopictus*	10	4
Negative	233	338
Total	276	372
Total	303	372

#### Mark-release-recapture

**Fig. 3:**
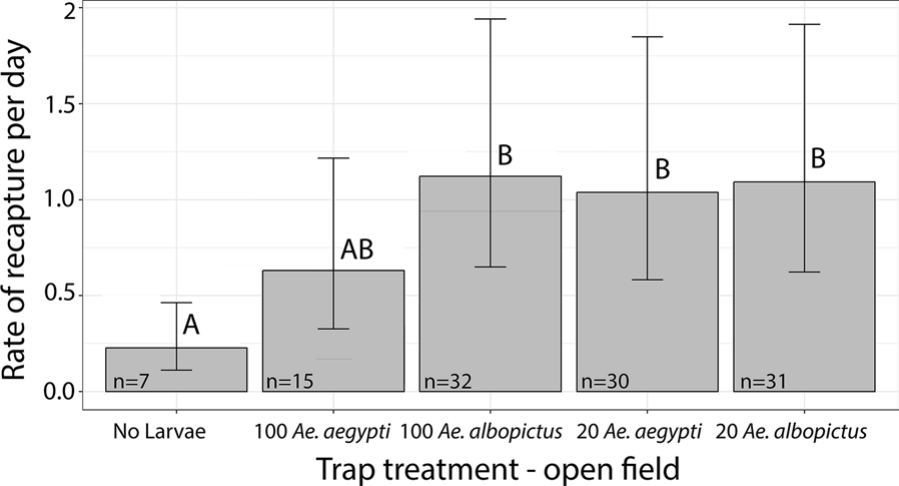
Results of the open-field experiment showing marked-released-recaptured *Ae. albopictus*. Mean recapture rates per day ± 95% confidence intervals are reverse transformed from the estimated marginal means of our Poisson distributed generalized mixed model. Significantly different treatments are indicated by letters. More *Ae. albopictus* were recaptured in traps with 100 *Ae. albopictus*, 20 *Ae. aegypti* and 20 *Ae. albopictus* than in traps with no larvae (*P* = 0.0006, *SE* = 0.0806; *P* = 0.0014, *SE* = 0.0875; *P* = 0.0008, *SE* = 0.082). The number of *Ae. albopictus* recaptured in traps with 100 *Ae. aegypti* was not significantly different than traps with any species/density combination or the control traps

The overall recapture rate was 12.85% and the total number recaptured was 115, which are numbers consistent with or higher than other studies using similar trapping methods [29, 30]. The number of marked *Ae. albopictus* recaptured in oviposition containers was affected by the presence of larvae in the ovitraps with significantly more *Ae. albopictus* recaptured in traps with larvae of either species than in traps with no larvae (*P* < 0.0001, *Z*-ratio = − 3.999, *SE* = 0.357). Significantly more *Ae. albopictus* were recaptured in traps with *Ae. albopictus* larvae at high densities (*P* = 0.0006, *Z*-ratio = − 4.653, *SE* = 0.0806) and in containers with *Ae. aegypti* larvae at low density (*P* = 0.0014, *Z*-ratio = − 3.801, *SE* = 0.0875) than in the no larvae control traps (Fig. 3). The number of *Ae. albopictus* recaptured in traps with high density *Ae. aegypti* was not significantly different than traps with any species/density combination (low density *Ae. aegypti*: *P* = 0.686, *Z*-ratio = − 0.169, *SE* = 0.608; low density *Ae. albopictus*: *P* = 0.594, *Z*-ratio = − 2.323, *SE* = 0.578) nor than the control traps (*P* = 0.156, *Z*-ratio = − 1.816, *SE* = 0.082) (Fig. 3). The number recaptured in ovitraps with low density *Ae. albopictus* was significantly higher than in the traps with no mosquito larvae (*P* = 0.0008, *Z*-ratio = − 4.204, *SE* = 0.082) (Fig. 3). All other variables had non-significant effects.

### Semi-field cage experiment

**Fig. 4:**
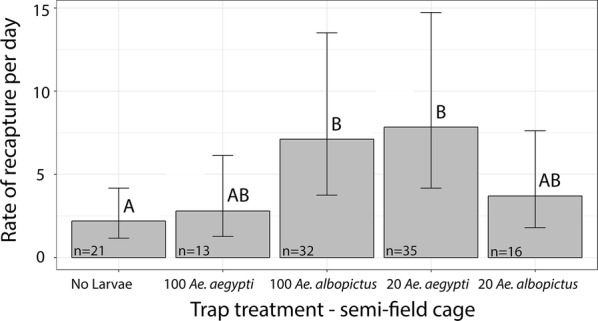
Results of the semi-field experiment showing released-recaptured *Ae. albopictus.* Mean recapture rates per day ± 95% confidence intervals are reverse transformed from the estimated marginal means of our Poisson distributed generalized mixed model. Significantly different treatments are indicated by letters. More *Ae. albopictus* were recaptured in traps with 100 *Ae. albopictus* and 20 *Ae. aegypti* than in traps with no larvae (*P* = 0.0349, *SE* = 0.127; *P* = 0.0161, *SE* = 0.115). The number of *Ae. albopictus* recaptured in traps with 100 *Ae. aegypti* and 20 *Ae. albopictus* was not significantly different than in traps with any species/density combination or the control traps

We recaptured a total of 117 females, for a recapture rate of 78.52%. The number of *Ae. albopictus* recaptured in oviposition containers was affected by the presence of larvae in the ovitraps (*P* = 0.0260, *Z*-ratio = − 2.072, *SE* = 0.403). Just as in the open-field experiment, significantly more *Ae. albopictus* were recaptured in containers with *Ae. albopictus* at high densities (*P* = 0.0349, *Z*-ratio = − 2.855, *SE* = 0.127) and in containers with *Ae. aegypti* larvae at low densities (*P* = 0.0161, *Z*-ratio = − 3.108, *SE* = 0.115) than in the control traps with no larvae (Fig. 4). Again, the number of *Ae. albopictus* recaptured in traps with high density of *Ae. aegypti* was not significantly different than in traps with any species/density combination (low density of *Ae. aegypti*: *P* = 0.186, *Z*-ratio = − 2.183, *SE* = 0.168; low density of *Ae. albopictus*: *P* = 0.982, *Z*-ratio = − 0.552, *SE* = 0.384, high density of *Ae. albopictus*: *P* = 0.282, *Z*-ratio = − 1.968, *SE* = 0.187) or than in the control traps (*P* = 0.986, *Z*-ratio = − 0.517, *SE* = 0.365) (Fig. 4). In this experiment, the number recaptured in the trap with low density of *Ae. albopictus* was also not significantly different than in the control (*P* = 0.775, *Z*-ratio = − 1.158, *SE* = 0.267) or the preferred traps (high density of *Ae. albopictus*: *P* = 0.598, *Z*-ratio = 1.446, *SE* = 0.871; low density of *Ae. aegypti*: *P* = 0.453, *Z*-ratio = 1.670, *SE* = 0.954) (Fig. 4). All other variables had non-significant effects.

## Discussion

This study addresses female *Ae. albopictus* oviposition choices that may lead to larval competition with *Ae. aegypti*. We tested attraction to ovitraps containing either conspecific larvae or heterospecific *Ae. aegypti* larvae at two levels of density in both the open-field and a semi-field cage. We showed that *Ae. albopictus* preferentially seek oviposition sites that contain larvae over those that do not, and that the combination of species and density of larvae in the container influenced oviposition site choice; those with high densities of conspecific larvae or low densities of heterospecific larvae were most attractive. Our results support previous laboratory studies and show that this behavior is relevant in the field.

Our semi-field cage and open-field experiments demonstrated that *Ae. albopictus* preferentially seek oviposition sites that contain larvae over those that do not. These results are corroborated by previous laboratory studies demonstrating that *Ae. albopictus* preferentially oviposit in sites that already contain larvae [18, 32–34]. This attraction is thought to be because the presence of conspecific larvae can indicate high-quality habitat; in *Ae. aegypti* it has been hypothesized to signal adequate food and infrequent desiccation [35]. We chose to use a consistent initial amount of larval nutrition (mean weight of 3.56 g per trap) rather than a set amount per larvae in the trap, which means that over the course of each replicate the nutrition was differentially depleted by larvae in each experimental treatment. However, if oviposition attraction was solely based on nutrient levels, we would expect the treatments with more larvae to be less attractive. We did not see this effect, further supporting our conclusion that larvae produce cues independent of nutrient levels that influence gravid female oviposition site selection.

We recaptured more gravid *Ae. albopictus* females in ovitraps with 100 *Ae. albopictus* than in traps with no larvae in both the open-field experiment and the semi-field cage experiment. Mosquitoes should seek oviposition sites that optimize offspring fitness, which is a balance between ensuring sufficient resources and minimizing detrimental competition. Although conspecific larvae can signal sight productivity, very high larval density lengthens development time and decreases adult mosquito size, thereby reducing fitness [34]. Therefore, attraction to conspecific larvae has been theorized to be hump-shaped density-dependent, with attraction increasing with greater numbers of larvae until a critical maximum, after which density is too high, and attraction declines. In an open-field study of *Ae. albopictus* oviposition site preference based on just conspecific larvae, preference increases with increasing numbers of larvae in the container until 130 larvae/0.37 l water, and decreased if larval density was greater [36]. This result is consistent with our findings that 100 conspecific larvae/3.7 l, a density level well before the critical maximum, was more attractive to *Ae. albopictus* adults than containers with no larvae.

More *Ae. albopictus* were recaptured in traps with 20 *Ae. aegypti* than with no larvae, but *Ae. albopictus* showed no preference for containers with 100 *Ae. aegypti* compared to larvae-free containers. There is an overlap in preferred larval environment for *Ae. aegypti* and *Ae. albopictus,* so the presence of heterospecific larvae may still be used to detect high-quality habitat, as is the case in other mosquito systems [37–39]. We suggest that, while heterospecific larvae may be attractive, the density tolerance threshold might be lower and dependent on existing resource levels. In our study, it may be that the combination of low numbers of heterospecific larvae with high resource surplus was attractive, but they had an intermediary preference towards containers with a greater number of *Ae. aegypti* larvae and a lesser surplus of nutrients. However, the results from previous studies are sparse and inconsistent. One laboratory study showed that *Ae. albopictus* prefers to oviposit in water used to rear *Ae. aegypti* [18]. A field study found that *Ae. albopictus* preferred water that had never contained larvae over water used to rear *Ae. aegypti* under non-stressful nutrient conditions [21]. Another laboratory study [19] found no attraction to “low” and “medium” (10 or 100 larvae/60 ml water) densities of *Ae. aegypti* larvae and strong preferential oviposition to “high” (500 larvae/60 ml water) densities compared to an ovicup with no larvae, but medium and high densities in this paper are far above those observed in the field.

The stark contrast between these three studies may be due to their methodological differences or to genetic variation between strains used. Each of these protocols varied in the larval densities tested, the use of water containing larvae *versus* strained larval rearing water, and if they were conducted in the laboratory *versus* in the field. It is also of note that all the previous studies used sieved water previously used for rearing larvae or placed washed larvae in clean water, which also contrasts with our methodology. Alternatively, the different results could be due to genetic variation between the mosquito strains. These species can undergo rapid local adaptation, and strain variation has been shown to be responsible for behavioral differences such as escape response to pesticides [40, 41], mating behavior [42, 43], and larval alarm reactions [44]. Furthermore, the interspecific competitive ability of *Ae. aegypti* against *Ae. albopictus* at the adult stage has been shown to vary by location. Strains of *Ae. aegypti* with previous exposure to *Ae. albopictus* are less susceptible to satyrization than those with minimal interspecific exposure [45]. It may be that susceptibility to larval competition varies by strain as well, which could consequently impact oviposition choice by strain. Future research could test a range of mosquito strains, and vary larval densities and resource levels to pinpoint their respective effects on oviposition preference.

It is unclear why there was a significant preference for traps containing 20 *Ae. albopictus* over the traps with no larvae in the open-field experiment, but no significant preference for or against this treatment in the semi-field cage experiment. If our results were to support the theory that attraction to conspecific larvae increases as the density increases until 130 larvae/0.37 l, we would expect our 100 *Ae. albopictus* treatment to be most attractive, our 20 *Ae. albopictus* treatment to be intermediately attractive, and our control to be least attractive. This was, in fact, the case in the semi-field cage, but not in the open-field. This may be because other environmental cues were present in the open-field setting, or because of an undetected effect of the bucket used.

The highly significant preference for containers with larvae with our sample size indicates that this preference is strong, and the similarity of the open-field and semi-field cage findings further corroborate our conclusions. Our results contradict one previous field study which found an idiosyncratic preference of *Ae. albopictus* for ovicups with con- and hetero-specific larvae [21]. This discrepancy may be due to methodological variation. While we used 5-day-old larvae in the ovitraps, they raised mosquitoes to eclosion and used sieved rearing water. These approaches reflect equally appropriate but different field conditions. Furthermore, their experimental containers were open-air, and they used total eggs laid as their response. Attraction to semiochemicals by mosquitoes often occurs over long distances, but any gustatory or tactile oviposition cues require contact with the water [46, 47]. Our ovitrap design did not permit females to touch water, and so our results only reflect preference for cues detected at a range. Further experimentation should compare these cues and their effect on active oviposition.

*Aedes aegypti* were not released in this study. The World Mosquito Programme is currently releasing *Wolbachia*-positive *Ae. aegypti* in Medellín and maintains a proprietary right on these mosquitoes. In order to conduct field trials with *Ae. aegypti*, researchers must use Medellín-origin *Ae. aegypti* that are confirmed negative for *Wolbachia*, and we were unable to create a compliant laboratory colony within our time frame.

## Conclusions

The invasion of Medellín, Colombia by *Ae. albopictus* is a recent and ongoing phenomenon. Any resulting competition with *Ae. aegypti* could preferentially favor one species over the other and alter the distribution or abundance of both species. This in turn could have implications on the spread of arboviral disease. Medellín is still predominantly inhabited by *Ae. aegypti,* and larval populations are low enough that ovipositing females can select between containers with or without other larvae. Because oviposition site-seeking *Ae. albopictus* actively are attracted to those with both conspecific and heterospecific mosquitoes, larval competition should be exacerbated as *Ae. albopictus* continues to invade. Further studies are needed to understand the impact of interspecific larval competition on the fitness of both species and to understand in more detail how ecological context modifies the outcome of oviposition behavior and interspecific competition.

## Data Availability

The datasets used and/or analyzed during the current study are available from the corresponding author on reasonable request.

## Abbreviation

SE: standard error
